# Generation Mechanism of Nonlinear Rayleigh Surface Waves for Randomly Distributed Surface Micro-Cracks

**DOI:** 10.3390/ma11040644

**Published:** 2018-04-23

**Authors:** Xiangyan Ding, Feilong Li, Youxuan Zhao, Yongmei Xu, Ning Hu, Peng Cao, Mingxi Deng

**Affiliations:** 1 College of Aerospace Engineering, Chongqing University, Chongqing 400044, China; ddingxiangyan@yeah.net (X.D.); feitian2302@sina.com (F.L.); aht2010@sina.com (Y.X.); dengmx65@yahoo.com (M.D.); 2 Key Laboratory of Optoelectronic Technology and Systems of the Education Ministry of China, Chongqing University, Chongqing 400044, China; 3 Department of Hydraulic Engineering, Tsinghua University, Beijing 100084, China; caopeng518888@126.com

**Keywords:** nonlinearity, Rayleigh surface waves, micro-cracks, numerical simulation

## Abstract

This paper investigates the propagation of Rayleigh surface waves in structures with randomly distributed surface micro-cracks using numerical simulations. The results revealed a significant ultrasonic nonlinear effect caused by the surface micro-cracks, which is mainly represented by a second harmonic with even more distinct third/quadruple harmonics. Based on statistical analysis from the numerous results of random micro-crack models, it is clearly found that the acoustic nonlinear parameter increases linearly with micro-crack density, the proportion of surface cracks, the size of micro-crack zone, and the excitation frequency. This study theoretically reveals that nonlinear Rayleigh surface waves are feasible for use in quantitatively identifying the physical characteristics of surface micro-cracks in structures.

## 1. Introduction

Stress corrosion micro-cracks, on or near a material surface, can susceptibly occur in metallic structures due to fatigue loads and corrosive environments [1,2]. Thus, it is of crucial importance to develop various non-destructive testing methods to detect and evaluate surface cracks for ensuring the integrity and safety of metallic structures. Unfortunately, it is well-known that linear ultrasonic methods based non-destructive testing technologies are not capable of detecting closed micro-cracks [3,4]. On the other hand, many studies have demonstrated that nonlinear ultrasonic-based non-destructive testing technologies are effective for evaluating early material degradation and micro-crack initiation by analyzing the nonlinear effects of ultrasonic waves [5,6,7,8,9,10,11,12].

The energy of Rayleigh surface waves is generally concentrated in the region near the surface within a depth of about one wavelength, and therefore, it is convenient and essential to utilize them to detect and evaluate surface cracks. In recent years, great efforts have been devoted to the detection of early material degradation using the higher harmonics of Rayleigh surface waves [13,14,15,16,17,18,19,20,21]. For instance, Achenbach et al. [13] have conducted an analytical far-field solution for the cumulative third harmonic surface wave, propagating on a half-space of isotropic incompressible cubically nonlinear material, in a relatively simple and systematic manner. Herrmann et al. [14] have reported a linear increase of the amplitude of a second harmonic surface wave using propagation distance by an experimental investigation.

It is supposed that when elastic waves reach a micro-crack, the crack can be closed and opened under compression and tension [22,23,24], and the compressive part of Rayleigh waves can penetrate through the crack while the tensile part cannot. The higher harmonics are generated due to the apparent local stiffness changes at the crack region under tension and compression. However, compared with works on early material degradation, research on the detection of micro-cracks using higher harmonics of Rayleigh surface waves is still rarely reported [6]. Oberhardt et al. [6] have modelled randomly distributed surface-breaking micro-cracks and investigated their relationship to second harmonic generation in Rayleigh surface waves with micro-crack density. In their study [6], a hyperelastic constitutive law was derived and implemented to simplify the micro-crack model. Rjelka et al. [25] have determined the relationship between material properties (third-order elastic constants) and inhomogeneous distributions of micro-cracks. They then investigated the acoustic nonlinearity parameter for surface acoustic waves based on second harmonic generation.

In this work, and different from Oberhardt et al. [6] and Rjelka et al. [25], the simplified micro-crack model is not used. We employ a true contact model for modelling the micro-cracks. Besides considering the normal contact stiffness, the Coulomb friction law for the tangential direction is adopted in the contact model, and the linear elastic law for material is employed. We focus on the mechanism of second harmonic generation and propagation of Rayleigh surface waves in metallic structures with randomly distributed surface micro-cracks. In addition, the quantitative relationships between the acoustic nonlinear parameter and the micro-cracks are also studied. This work will provide a theoretical basis for detection and evaluation techniques based on nonlinear Rayleigh surface waves.

## 2. Acoustic Nonlinearity Parameter for Rayleigh Waves

To date, a substantial number of experiments have demonstrated that the nonlinear ultrasonic detection technique, based on the second harmonics of bulk waves, Lamb waves, and Rayleigh surface waves, is effective for detecting changes to the microstructure of metal materials [26]. The amplitude of the second harmonic waves can be quantitatively evaluated using the acoustic nonlinearity parameter.

Considered a weak quadratic nonlinearity, the acoustic nonlinearity parameter of Rayleigh surface waves at the surface $\bar{u_{y}}=u_{y}\;\left(y=0\right)$ can be expressed as [14,18,26]:

(1) $$\beta =\frac{\bar{u_{y}}\left(2\omega \right)}{{\bar{u}}_{y}^{2}\left(\omega \right)x}\;\frac{i8p}{\kappa_{P}^{2}\kappa_{R}}\left(1-\frac{2\kappa_{R}^{2}}{\kappa_{R}^{2}+s^{2}}\right)$$

where $p^{2}=\kappa_{R}^{2}-\kappa_{P}^{2}$, $s^{2}=\kappa_{R}^{2}-\kappa_{S}^{2}$, $\kappa_{R}$, $\kappa_{P}$, and $\kappa_{S}$ are the wavenumbers for the Rayleigh, longitudinal, and shear waves, respectively.

However, the contact acoustic nonlinearity caused by cracks is different from classical acoustic nonlinearity. Therefore, a different acoustic nonlinearity parameter $\beta^{\prime }=A_{2}/A_{1}$ is used in this study [22,23,24], where *A*_1_ and *A*_2_ are, respectively, the amplitudes of the fundamental and second harmonic waves. This parameter is related to the crack density, the frequency of fundamental waves, the propagation distance, and the friction coefficient.

## 3. Numerical Simulation

Since it is a challenge to directly obtain the nonlinear response when a Rayleigh wave passes through micro-cracks by an analytic method, numerical simulations are employed here. A two-dimensional finite element method (FEM) model of a structure with randomly distributed micro-cracks is built using the commercial FEM software ABAQUS (Version 6.14) and Python (Version 2.7).

**Figure 1:**
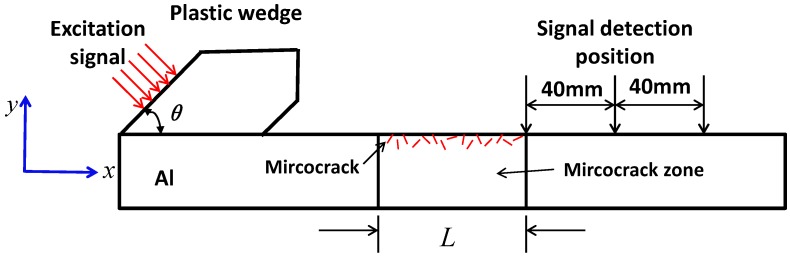
Schematic of Rayleigh surface waves propagating through a region with micro-cracks.

The problem of Rayleigh surface waves propagating through a structure with surface micro-cracks is described in Figure 1. The micro-cracks are at or near the surface of the middle region of the specimen, which is named as a micro-crack zone with length *L* (Note that the range of *L* in this paper is from 10 mm to 30 mm). In order to excite Rayleigh surface waves, longitudinal waves are applied using a dynamic displacement excitation on the flank of the wedge. Rayleigh waves are then excited due to the Rayleigh critical angle *θ*. These waves propagate along the positive direction of *x*, and interact with the micro-cracks when they reach the micro-crack zone. As a result, the second harmonic waves are generated. Because the size of micro-cracks is much smaller than the wavelength of Rayleigh surface waves, most fundamental waves pass through the micro-crack zone and are collected at three signal detection positions (named as transmitted waves), which are 0 mm, 40 mm, and 80 mm away from the right boundary of the micro-crack zone, respectively, as shown in Figure 1.

To investigate the influence of micro-cracks on the acoustic nonlinearity parameter, *N* micro-cracks are randomly distributed at or near the surface of the middle part with length *L*. Note that when micro-cracks are near the surface, the distances from the surface to the centers of micro-cracks are less than the wavelength of the Rayleigh wave. The parameter *w*% is defined as the ratio of the number of surface micro-cracks divided by the total number of micro-cracks, which represents the proportion of micro-cracks on the surface. Thus, w% = 100% means that all the micro-cracks are on the surface. Additionally, all the cracks have the same length of 2*a* (the range of *a* is from 25 µm to 75 µm in this study). Therefore, the crack density can be defined as *c* = *Na/L*, where *c* is a dimensionless parameter. In this study, there are two variables that affect the random distribution of micro-cracks, i.e., the center position and the orientation of cracks, which are described by the probability density function (PDF) with a uniform random variable in the simulations.

A linear elastic constitutive law of aluminum (AL-6061-T6) for the specimen and plastics for the wedge are considered in the FEM analysis, and the material properties used in the simulations are $\rho_{\mathrm{Al}}$ = 2704 kg/m^3^, $E_{\mathrm{Al}}$ = 68.9 GPa, $v_{\mathrm{Al}}$ = 0.33, $\rho_{\mathrm{Plastic}}$ = 1180 kg/m^3^, $E_{\mathrm{Plastic}}$ = 4 Gpa, and $v_{\mathrm{Plastic}}$ = 0.33. Thus, the Rayleigh critical angle *θ* = 53.28° can be calculated by the material properties of aluminum and plastics.

**Figure 2:**
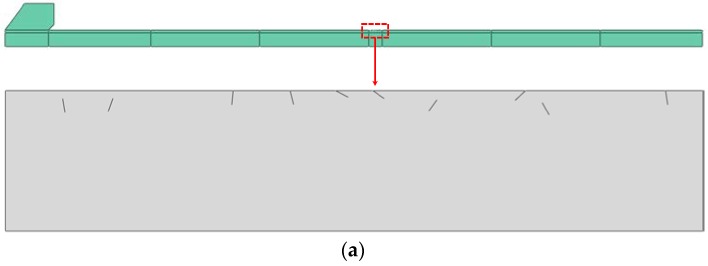

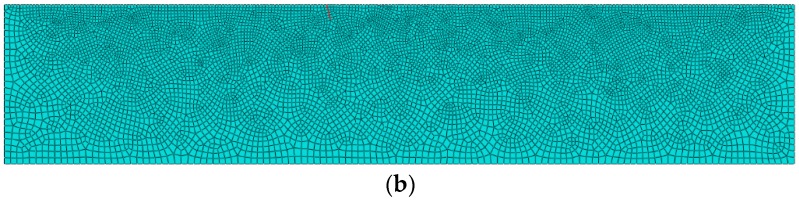
FEM model of the micro-crack zone: (**a**) distribution of micro-cracks; (**b**) finite element mesh (the red line represents one crack in the micro-crack zone).

The size of the FEM model is 240 × 6 mm. Since at least 20 elements are required in the wavelength of the highest excitation frequency waves (2000 kHz in this study), from the aspect of numerical accuracy, at least 6 elements for each crack are employed with certainty, and the element size for the non-crack zone is set to *L*_max_ = 0.08 mm. The FEM model is constructed using the four-node plane strain (CPE4R) elements, as shown in Figure 2.

A micro-crack is modelled as two contact surfaces, which can be separated from, but cannot interpenetrate into, each other. In addition, the Coulomb friction law is employed to model the relative sliding of the surfaces, and the friction coefficient is *µ*. Thus, the actual traction applied to the crack faces is given by [8]:

(2) $$q=\sigma_{0}n+\left[\tau_{0}+\mu \sigma_{0}H\left(-\sigma_{0}\right)\right]H\left[\tau_{0}+\mu \sigma_{0}H\left(-\sigma_{0}\right)\right]s$$

where $\sigma_{0}$ and $\tau_{0}$ are, respectively, the normal and shear stresses on the surfaces of the crack, H is the Heaviside step function, **n** is a unit vector that is perpendicular to the crack face, and **s** is a unit vector in the plane of the crack face.

The dynamic displacement excitation applied to the flank of the wedge can be expressed as $u\left(x,t\right)=A_{0}\sin\left(2\pi ft\right)\times \sin{\left(\pi ft/10\right)}^{2}$, where $A_{0}$ is the amplitude of excitation signal (1 × 10^−4^ mm in this study [8,27,28]) and *f* is the frequency of the excitation signal (ranging from 800~2000 kHz). In addition, the frequency needs to be low enough to satisfy the low frequency assumption, leading to *λ*/*a* ≈ 20, where *λ* is the wavelength and *a* is the half-crack length [24]. Furthermore, the non-reflecting or absorbing boundary conditions at the bottom and right edges of the FEM model are used to maximally eliminate the influence of boundary reflection.

In this study, a ABAQUS/Explicit solver, based on the central different method, is employed to simulate the propagation of Rayleigh surface waves in the time domain, which is conditionally stable. The smallest time increment for the current problem that leads to a stable solution is Δt = 1 × 10^−9^ s. However, considering the higher harmonic waves generated by the interactions between the excitation signals and micro-cracks, the time increment is set to be Δt = 2 × 10^−10^ s, to be on the safe side. In addition, a double precision operation is used to reduce the accumulative error here.

**Figure 3:**
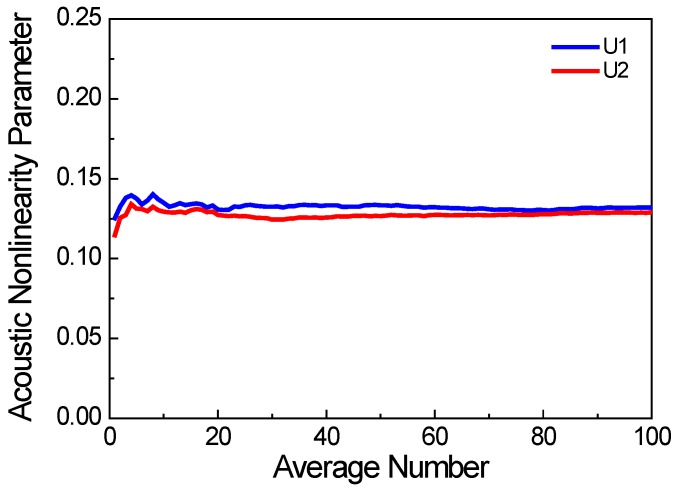
Acoustic nonlinearity parameter versus number of FEM models (*c* = 0.1, *f* = 1000 kHz, *µ* = 0.1, w% = 60%).

Moreover, an additional consideration is the randomness of the crack distribution. To capture the stochastic nature of the random distribution, multiple FEM models are constructed with different realizations of the random distribution. Figure 3 shows the average acoustic nonlinearity parameter versus the number of FEM models (U1 and U2 are the displacements in the *x* and *y* direction, respectively), where *c* is the crack density, *f* is the excitation frequency, *µ* is the friction coefficient of the micro-crack surfaces, and w% is the proportion of surface cracks. The acoustic nonlinearity parameter is more stable when the number of FEM models is larger than 50. Therefore, the results shown in the following section are averaged over 100 FEM models.

## 4. Result and Discussion

When Rayleigh surface waves pass through micro-cracks, they can make the crack faces open under tension and closed under compression, which generates acoustic nonlinearity [24,29]. Meanwhile, because the wavelength *λ* (1.4–3.6 mm) of Rayleigh surface waves is much larger than the crack length (50 µm to 150 µm), most of the waves can pass through the micro-crack zone. Furthermore, to investigate quantitative relationships between the acoustic nonlinear parameter and the micro-cracks, some representative characteristics or parameters, i.e., the crack density, the propagation distance in the micro-crack zone, the proportion of surface cracks (i.e., w%), the frequency of the fundamental wave, and the friction coefficient of the micro-crack surfaces, are considered here.

**Figure 4:**
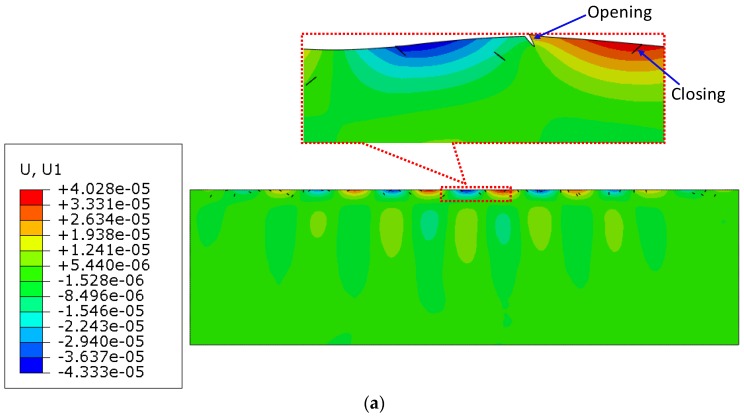

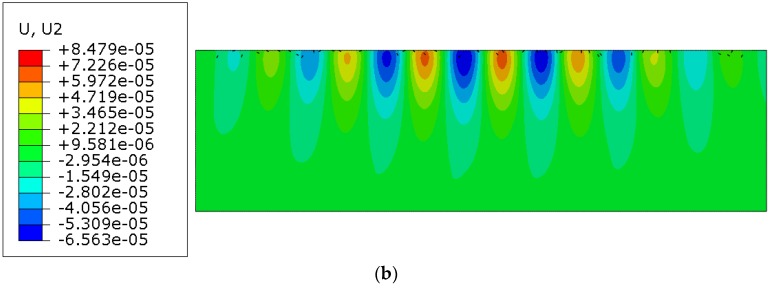
Propagation of surface waves in a structure with surface micro-cracks (*c* = 0.1, *f* = 1000 kHz, *µ* = 0.1, w% = 60%). (**a**) Displacement contour in the *x* direction (800 times zoomed deformed shapes in the dashed box); (**b**) Displacement contour in the *y* direction.

Figure 4 shows the displacement contour of Rayleigh surface waves while propagating in a structure with surface micro-cracks at 1000 kHz. We can see clearly that the wavelength of Rayleigh surface waves is much larger than the crack length. Moreover, although the waves interact with the micro-crack surfaces in the micro-crack zone, no obvious change is observed in the waveforms. Only a very weak scattering phenomenon occurred around the micro-cracks. Meanwhile, the 800 times zoomed deformed shapes clearly demonstrate the opening and closing states of the micro-cracks.

**Figure 5:**
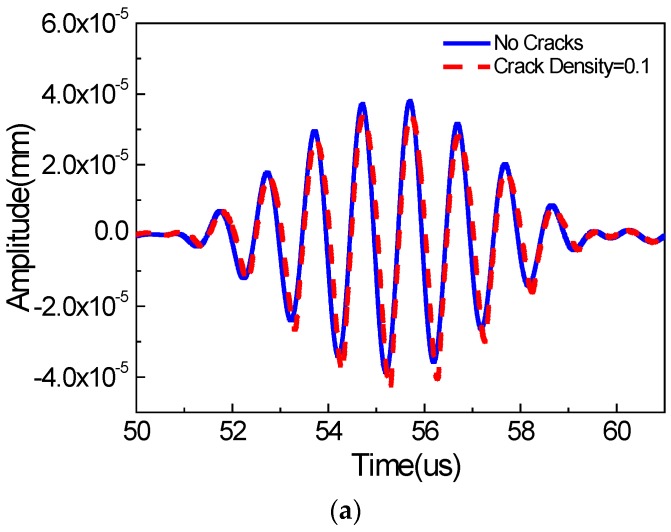

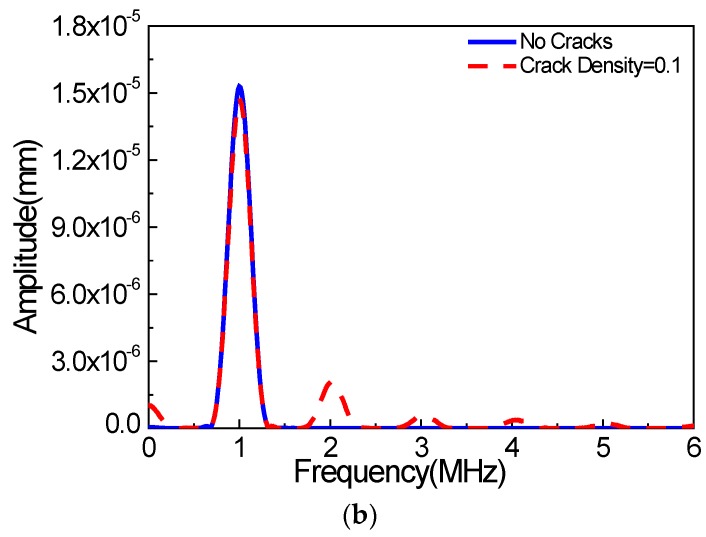
Wave signals in the *x* direction (**a**) time domain; (**b**) frequency domain (*c* = 0.1, *f* = 1000 kHz, *µ* = 0.1, w% = 60%).

**Figure 6:**
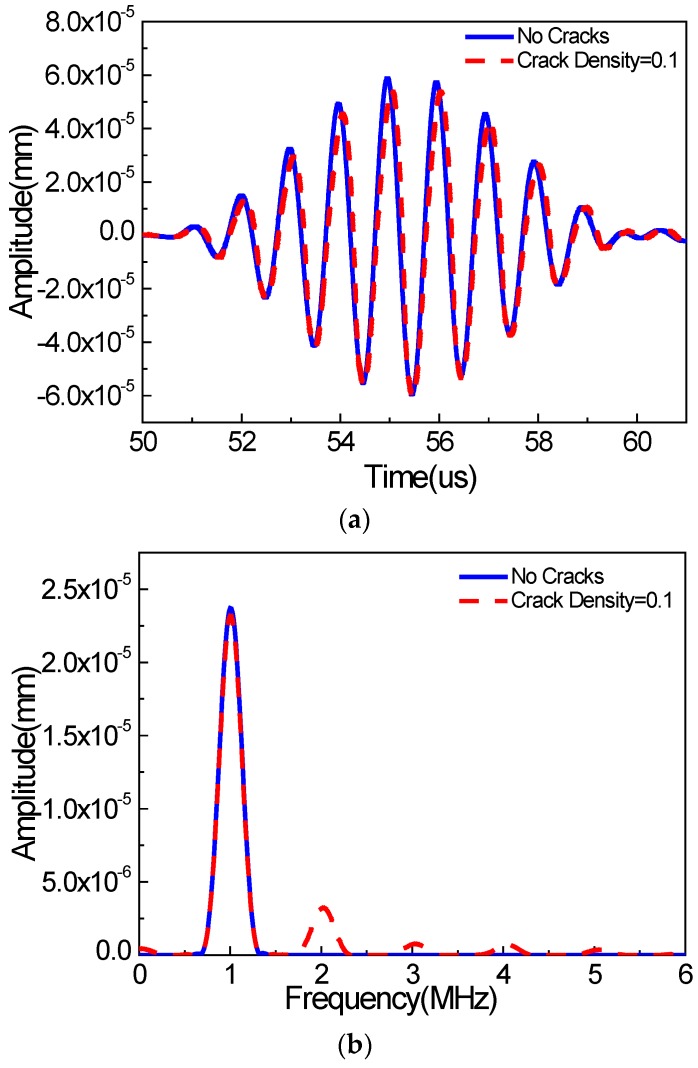
Wave signals in the *y* direction (**a**) time domain; (**b**) frequency domain (*c* = 0.1, *f* = 1000 kHz, *µ* = 0.1, w% = 60%).

Figure 5 and Figure 6 show wave signals collected at the signal detection position (0 mm away from the right boundary of the micro-crack zone) in the *x* and *y* directions, respectively, wherein the wave signals for the non-cracked case are marked by the solid line, and the wave signals for the cracked case are marked by the dashed line. We can see that the energies of the wave signals in the *x* and *y* directions are almost identical. Moreover, the time-domain signals for the non-cracked case and the cracked case in the *x* and *y* directions are very similar, as shown in Figure 5a and Figure 6a, respectively. More obvious differences can be observed from the frequency analysis of the wave signals (Figure 5b and Figure 6b). We find that the wave signals for the non-cracked case marked by the solid line only contain the 1000 kHz wave signals (the fundamental waves), indicating that the second harmonic waves cannot be generated for the non-cracked case. However, the wave signals for the cracked case, marked by the dashed line, contain not only the 1000 kHz wave signals, but also the 2000 kHz wave signals (second harmonic waves) and even distinct 3000/4000 kHz wave signals (third/quadruple harmonic waves), implying that the existence of micro-cracks is the critical factor for generating the second harmonic waves. Meanwhile, the third and quadruple harmonic waves for the case of Rayleigh surface waves are more remarkable compared to the cases of bulk [24] and Lamb [8] waves. Here, from numerical simulations, we find that, when using Rayleigh surface waves, micro-cracks could generate the second harmonic waves. Therefore, this study provides a foundation for some possible new techniques for identifying surface micro-cracks in a structure using Rayleigh surface waves.

**Figure 7:**
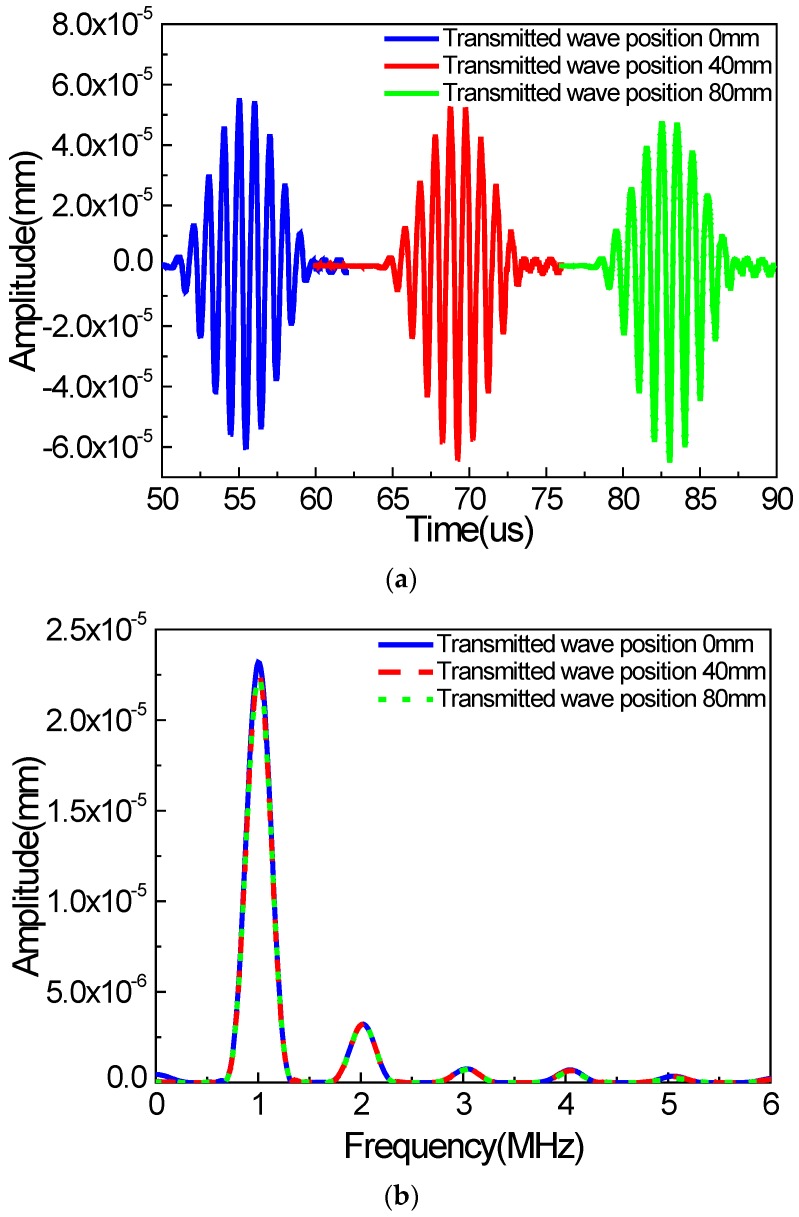
Waveforms of wave signals in the *y* direction at different locations (**a**) time domain; (**b**) frequency domain (*c* = 0.1, *f* = 1000 kHz, *µ* = 0.1, w% = 60%).

Next, the wave signals collected at 0 mm, 40 mm, and 80 mm, away from the right boundary of the micro-crack zone, are shown in Figure 7 (in the *y* direction). We can find that the waveforms of Rayleigh surface waves collected at different locations are the same, indicating that the second harmonics from the received signal are independent from the signal detection position. And the same tendency can be found in the *x* direction. If there exists damping, the amplitudes of the fundamental waves and the second harmonics could be reduced, but nevertheless, the acoustic nonlinear phenomenon cannot be affected significantly.

**Figure 8:**
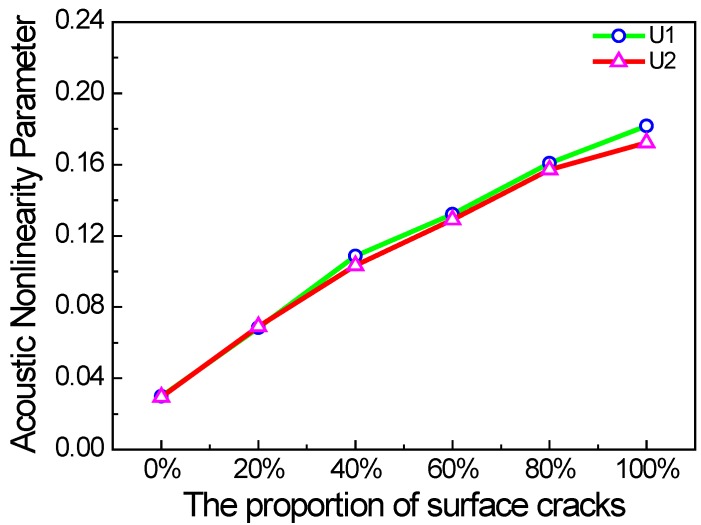
Acoustic nonlinearity parameter versus the proportion of surface cracks (*c* = 0.1, *f* = 1000 kHz, *µ* = 0.1).

Furthermore, we investigate the relationship between the acoustic nonlinearity parameter and the proportion of surface micro-cracks, as well as the crack density and propagation distance in the micro-crack zone. The acoustic nonlinearity parameter increases almost linearly with the proportion of surface micro-cracks as shown in Figure 8. When all the micro-cracks are on the surface (w% = 100%), the acoustic nonlinearity parameter reaches the maximum, which is the most serious case in practice.

**Figure 9:**
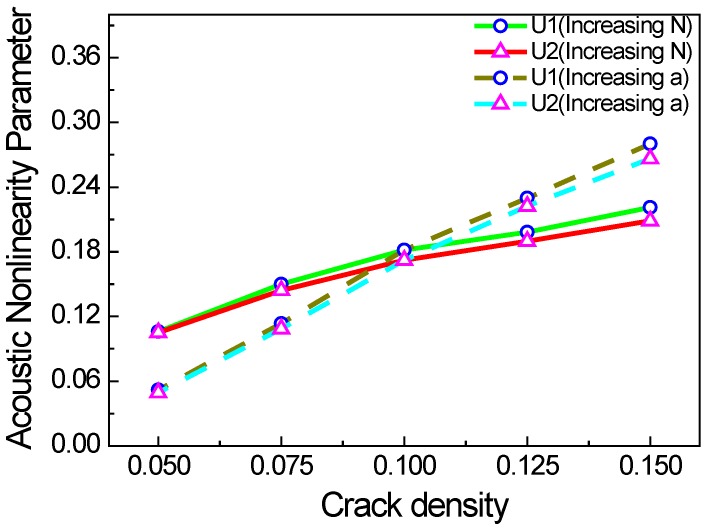
Acoustic nonlinearity parameter versus the crack density (*f* = 1000 kHz, *µ* = 0.1, w% = 100%).

Figure 9 shows the acoustic nonlinearity parameter versus the crack density *c*. The crack density (*c = Na/L*) is a dimensionless number, which can be used as a quantitative indicator for acoustic nonlinearity caused by micro-cracks. Because the crack density can be affected by parameters *N* and *a*, we consider the following two cases: the first case is to increase *N* and to keep *a* the same, and the second is to increase *a* and to keep *N* the same. When the excitation frequency and the friction coefficient of the micro-crack surfaces remain unchanged, the acoustic nonlinearity parameter is more sensitive to crack length than crack number, and the acoustic nonlinearity parameter increases linearly with the increase in crack density. We also find that the acoustic nonlinearity parameters in the *x* and *y* directions are consistent.

**Figure 10:**
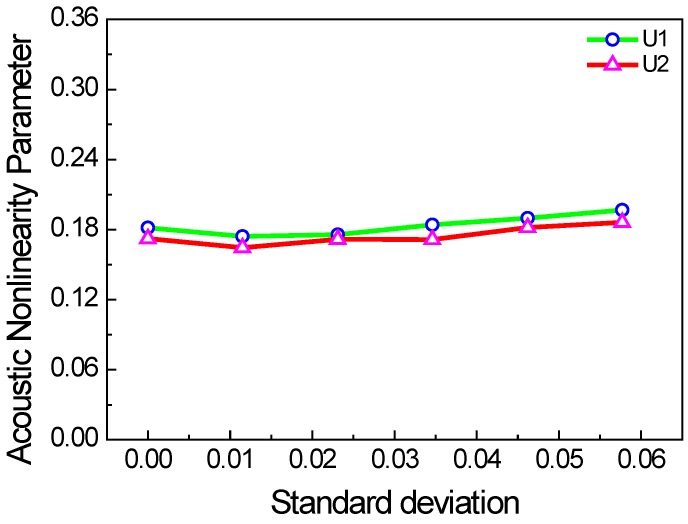
Acoustic nonlinearity parameter versus standard deviation of micro-crack lengths with uniform distribution (*c* = 0.1, *f* = 1000 kHz, *µ* = 0.1, w% = 100%).

**Figure 11:**
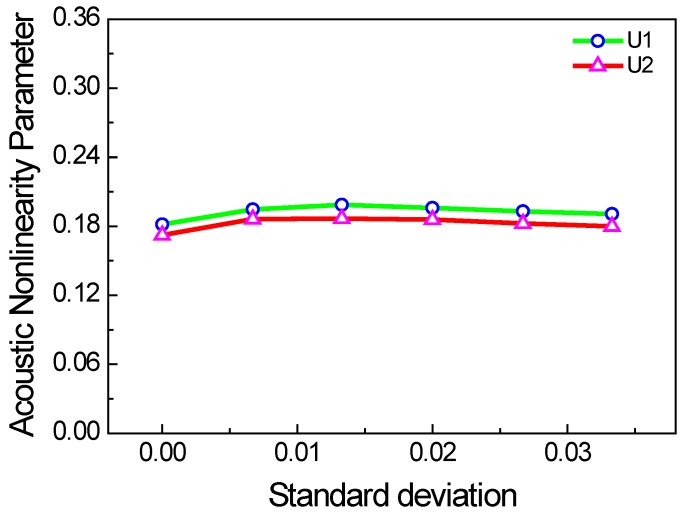
Acoustic nonlinearity parameter versus standard deviation of micro-crack lengths with Gaussian distribution (*c* = 0.1, *f* = 1000 kHz, *µ* = 0.1, w% = 100%).

The lengths of each micro-crack in practice should be different. Thus, we also consider the two cases with the crack length of uniform and Gaussian random distributions. In the two cases, the mean values are the same, but the standard deviations are changed. Figure 10 and Figure 11 show the acoustic nonlinearity parameter versus the standard deviation of micro-crack lengths. It is shown that the acoustic nonlinearity parameter does not change with standard deviation, which is only related to the mean value.

**Figure 12:**
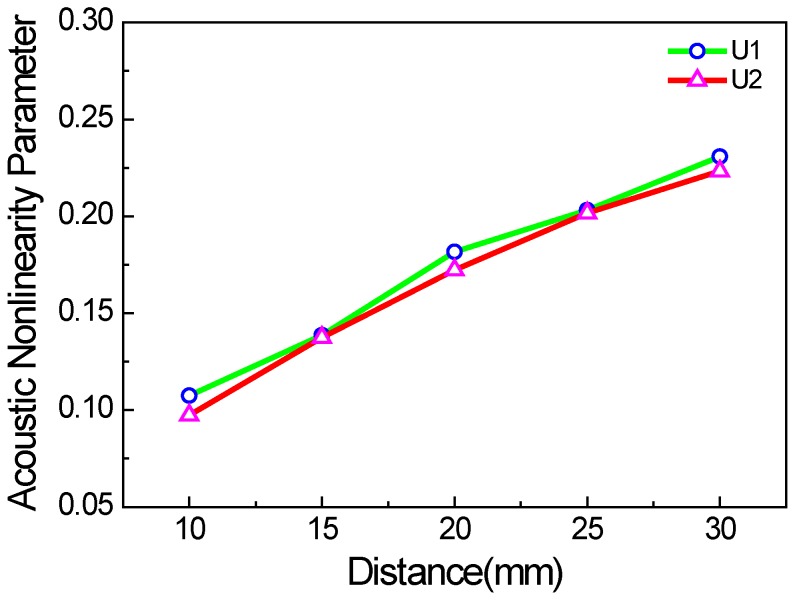
Acoustic nonlinearity parameter versus the propagation distance in the micro-crack zone (*c* = 0.1, *f* = 1000 kHz, *µ* = 0.1, w% = 100%).

Figure 12 shows the acoustic nonlinearity parameter versus the propagation distance in the micro-crack zone. The acoustic nonlinearity parameter also increases linearly with the propagation distance in the micro-crack zone, leading to its linear accumulation property.

**Figure 13:**
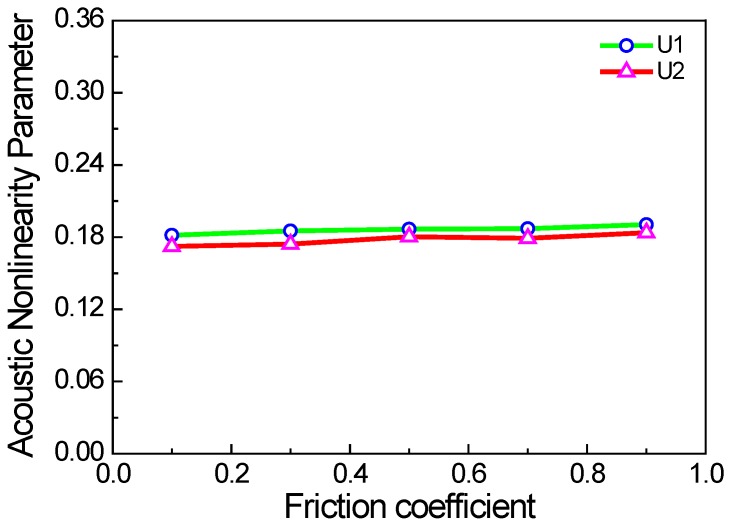
Acoustic nonlinearity parameter versus the friction coefficient (*c* = 0.1, *f* = 1000 kHz, w% = 100%).

**Figure 14:**
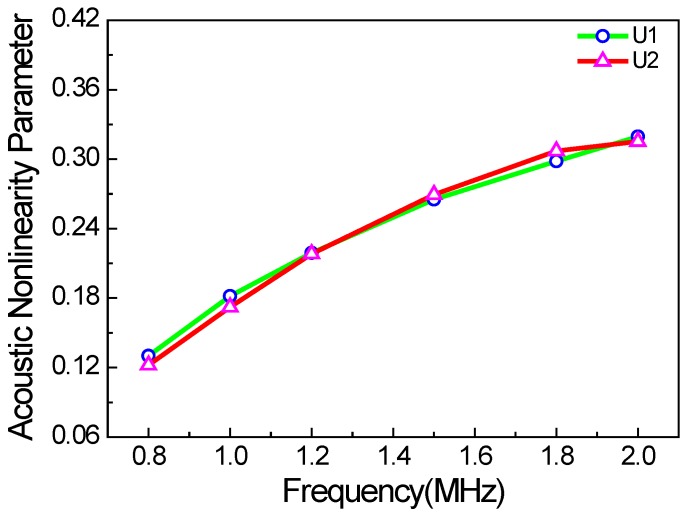
Acoustic nonlinearity parameter versus the excitation frequency (*c* = 0.1, *µ* = 0.1, w% = 100%).

In contrast to classic acoustic nonlinearity, the factors affecting acoustic nonlinearity also include excitation frequency and the friction coefficient of micro-crack surfaces. In this study, the Coulomb law of friction is used to calculate the frictional force between the micro-crack surfaces. When the crack density and the excitation frequency remain unchanged, the acoustic nonlinearity parameter has almost no relation to the friction coefficient, as shown in Figure 13. It should be noted that the acoustic nonlinearity parameter of the *x* direction is slightly higher than that of the *y* direction. The reasons could be the more pronounced clapping behavior of the cracks than the frictional one, and asymmetry due to the random micro-cracks modelling. Thus, it could be inferred that the clapping behavior of the cracks is more remarkable than the frictional one under the condition of randomly distributed micro-cracks. Figure 14 shows the acoustic nonlinearity parameter versus the excitation frequency. It can be seen that the acoustic nonlinearity parameters of the *x* and *y* directions increase linearly with the excitation frequency. The main reason is that the wavelength of the Rayleigh surface wave decreases when the excitation frequency increases, and the wave with a smaller wavelength is more sensitive to the micro-crack. Therefore, the nonlinear interactions between Rayleigh surface waves with a smaller wavelength and the micro-cracks became stronger, and the second harmonic was more noticeable.

**Figure 15:**
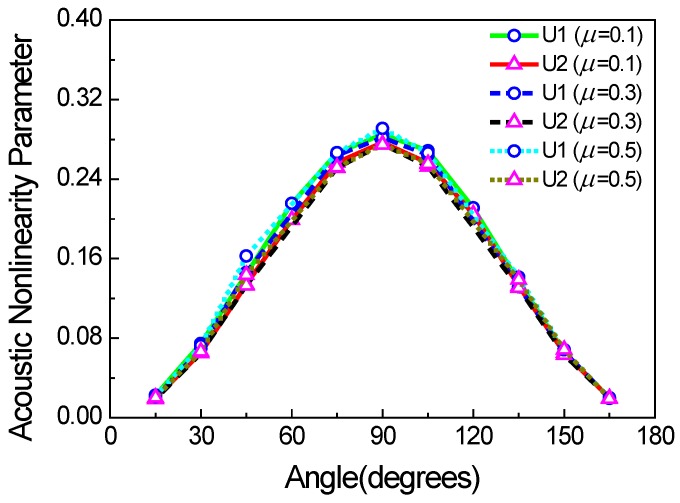
Acoustic nonlinearity parameter versus the angle of micro-cracks (*c* = 0.1, *f* = 1000 kHz, w% = 100%).

The above cases are all based on the assumption that crack angles have a uniform and random distribution with a range from 0° to 180° (the angle between the crack face and the *x* direction). However, the angles of micro-cracks [22,23] can affect the acoustic nonlinearity parameter. In this case, we consider that all the micro-crack angles are uniform, and the positions of micro-cracks are also of a uniformly random distribution. The acoustic nonlinearity parameter is shown as a function of the angle of micro-cracks in Figure 15. When the angle is 90°, the acoustic nonlinearity parameter is at the maximum, which is essentially caused by the considerable clapping behavior of the cracks rather than the frictional one. Meanwhile, we also find that the friction coefficient slightly affects the acoustic nonlinearity parameter under the condition of regularly arranged micro-cracks. The reason could be the weak energies of Rayleigh waves, which hardly generate the slipping trend between the surfaces of micro-cracks. Therefore, we can also infer that the clapping behavior is of more importance than the frictional one under the condition of regularly arranged micro-cracks.

## 5. Conclusions

A numerical model containing randomly distributed surface micro-cracks is constructed to investigate the propagation phenomenon of Rayleigh surface waves by statistical analysis from numerous results from random micro-crack models. The following paragraphs outline the main conclusions drawn from this study.

Firstly, when Rayleigh surface waves are used as the fundamental waves, the ultrasonic nonlinear effect, in terms of second harmonics, can be generated by surface micro-cracks. It is found that the third and quadruple harmonics of Rayleigh surface waves are more noteworthy than those of bulk and Lamb waves.

Second, the different distributions of the lengths and angles of surface micro-cracks were investigated. The results show that the acoustic nonlinearity parameter is only related to the mean value of the lengths of surface micro-cracks, and the acoustic nonlinearity parameter is at maximum when the angle of the surface micro-crack is 90°.

Finally, we systematically investigated the relationships between the acoustic nonlinear parameter and some key factors, i.e., the micro-crack density, the propagation distance in the cracking region, the proportion of surface cracks, the excitation frequency, and the friction coefficient of the micro-crack surfaces. The results reveal that the acoustic nonlinear parameters in the *x*-axis and *y*-axis are basically consistent, which is linearly proportional to the crack density, the proportion of surface cracks, the propagation distance of surface waves in the cracking region, and the excitation frequency. However, it is not correlated with the friction coefficient of the micro-crack surfaces. Therefore, the acoustic nonlinear parameter can be used to effectively characterize the degeneration of material properties caused by surface micro-cracks using the Rayleigh surface wave technique. Furthermore, a proper increase of the fundamental frequency can generate more significantly higher harmonics and improve the accuracy of this technique. These quantitative relationships provide the foundation for developing nonlinear ultrasonic based quantitative non-destructive evaluation techniques for assessing surface micro-cracks.
